# The fatty acid composition in follicles is related to the developmental potential of oocytes up to the blastocyst stage: a single-centre cohort study

**DOI:** 10.1186/s12958-022-00974-7

**Published:** 2022-07-25

**Authors:** Yujie Liu, Kelly Tilleman, Bruno Vlaeminck, Rachel Gervais, P Yvan Chouinard, Petra De Sutter, Veerle Fievez

**Affiliations:** 1 Department of Animal Sciences and Aquatic Ecology, Ghent University, Coupure Links 653, 9000 Ghent, Belgium; 2 Department for Reproductive Medicine, Ghent University Hospital, C. Heymanslaan 10, 9000 Ghent, Belgium; 3 Department of Biology, Ghent University, Krijgslaan 281, S8, 9000 Ghent, Belgium; 4 Department of Animal Sciences, Laval University, 2425, rue de l’Agriculture, Québec, Québec G1V 0A6 Canada

**Keywords:** Assisted reproductive technology, Body mass index, Age, Follicular fluid, Fatty acid, Blastocyst, Embryo

## Abstract

**Background:**

Advanced maternal age and obesity are associated with impaired female fertility. Moreover, fatty acids (FA) in follicular fluid (FF) play important roles in oocyte maturation and embryo development. However, the effects of body mass index (BMI), age, and FF FA composition on embryo development between days 3 and 5 and blastocyst stage on day 5 are still unclear.

**Methods:**

This study included 138 patients undergoing assisted reproductive technology (ART), which were divided into three BMI groups (18.5–24.9 kg/m^2^ vs. 25.0–29.9 kg/m^2^ vs. ≥ 30.0 kg/m^2^) and three age-related groups (20–30 years vs. 31–34 years vs. ≥ 35 years) which were compared for ART outcomes. Further, observations were divided into quartiles based on either of three parameters related to embryo outcome, i.e. (i) embryos developing between days 3 and 5 (ED3-5) and (ii) expanded blastocysts on day 5 (EB5), both expressed proportionally to the number of oocytes with two pronuclei (2PN), as well as (iii) the embryo utilization rate (EUR). Proportions of FF FA were then compared between Q1 and Q4, representing the quartile with the worst vs. the best embryo outcome, respectively. Finally, regression models were created to assess the relationships between BMI, age, FF total FA (TFA) concentration, relative proportions of specific FA and embryo outcome.

**Results:**

Patients of Q1 had higher proportions of FF C20:5n-3, C22:6n-3 and total n-3 PUFA than Q4 patients. Furthermore, Q4 patients tended to be younger than Q1 patients. Within the whole cohort, the proportion of C20:5n-3 negatively correlated with ED3-5/2PN and EUR, while EB5/2PN tended to be negatively correlated with age. Regression models within the overweight and obese group confirmed the negative relation between C20:5n-3 and ED3-5/2PN, but also indicated additional associations: C18:1n-9 and C20:4n-6 were positively associated with ED3-5/2PN and EUR, respectively while the proportion of C18:0 was negatively associated with EUR.

**Conclusion:**

The proportions of n-3 PUFA, particularly C20:5n-3 and C22:6n-3 were reduced in the patients’ quartile with the best embryo outcome. This group of patients was also younger. However, the embryo quality parameters of overweight/obese patients were not associated with age but were positively associated with FF C18:1n-9 and negatively with the proportions of C18:0 or C20:5n-3.

**Trial registration:**

This study’ registration number was B670201627735.

**Supplementary Information:**

The online version contains supplementary material available at 10.1186/s12958-022-00974-7.

## Introduction

Worldwide, a growing number of women are seeking assistance to get pregnant through assisted reproductive technology (ART) [1]. Delaying childbearing to later in life is one reason for the increased number of women of advanced maternal age opting for ART treatment [2]. Moreover, the epidemic of overweight and obesity in many European countries has resulted in nearly 30% of women in the reproductive age group to have excess adiposity [3], while obesity has been associated with reduced fecundity [4]. As a result, more overweight and obese women are relying on ART [3].

With regards to the ART outcome, several meta-analyses suggested a negative association between female obesity and live birth rates [5–7], while no adverse effects of obesity on pregnancy or live birth outcome have been reported in others [8, 9]. Although embryo quality largely determines the success of implantation and live birth [10], Herbemont et al. [11] indicated that poor or good embryo quality at days 2 and 3 were not predictive of the implantation of good-quality blastocysts. On the other hand, an increase in live birth rate was associated with blastocyst transfer on day 5 when compared with embryo transfer on day 3 [10]. Interestingly, the study by Blank et al. [12] revealed that pregnancy outcome could be enhanced when transferring an embryo that has an improvement in quality between days 3 and 5 as opposed to one that has remained stable. Accordingly, they recommended considering a dynamic evaluation of the embryo between days 3 and 5 when qualitatively equally scored (excellent) blastocysts were available on day 5 [12]. To the best of our knowledge, the kinds of environmental factors in the follicle that could be associated with differences in embryo development stage between days 3 and 5 and quality on day 5 are unknown.

As follicular fluid (FF) surrounds the maturing oocyte prior to fertilization, its characteristics can influence the outcome of assisted reproduction in terms of fertilization success, embryonic development, and pregnancy rate [13]. In FF, fatty acids (FA) play important roles as cellular energy sources, structural components of membranes, and precursors for prostaglandins and steroid synthesis [14]. In the study of O’Gorman et al. [15]*,* non-cleaved oocytes (which fertilized normally but failed to cleave) showed an increased proportion of C16:0 in FF compared with oocytes which developed into early cleavage stage embryos, while the proportions of C18:0, C20:4n-6 and C22:6n-3 were lower. Ruiz-Sanz et al. [13] showed that the levels of total n-3 polyunsaturated FA (PUFA), particularly C22:6n-3, as well as C20:4n-6 were significantly increased while lower levels of C18:3n-3 were noted in the FF of large compared to small follicles. However, investigations into the effect of FA available in the follicular environment on embryo development until day 5 are still lacking. Moreover, FF FA composition shows links with maternal age [13] and BMI [14]. Accordingly, we hypothesized that ART outcome might be mutually affected by these potentially confounding factors. Nevertheless, several former studies relating BMI to FF FA and ART outcome relied on a relatively limited number of patients, e.g. Valckx et al. [14] included 30 women equally distributed among three BMI groups (normal weight, overweight and obese), while in the research by Leary et al. [16], 29 patients were monitored (normal weight, *n* = 12; overweight + obese, *n* = 17). Accordingly, the possibility to mutually study potentially confounding factors in such studies is limited.

Therefore, the current study, including a reasonably large number of participants who were divided over three BMI classes and three age-related groups, as well as including a detailed analysis of FA in FF, aimed to evaluate the relation between BMI, age, FF FA composition and ART outcome, with particular focus on embryo development between days 3 and 5 and blastocyst stage on day 5.

## Materials and methods

### Participants and sample size of the cohort

During the sampling period from 2016 to 2018, 147 patients seeking assisted reproductive services at Ghent University Hospital (Ghent, Belgium) were enrolled in this study. The protocol was approved by the local ethics committee of the Ghent University Hospital (2016/0259, Belgian registration number: B670201627735). Informed consent was obtained from each participant prior to ART treatment. Patients were treated with either an agonist or antagonist protocol, followed by ovum pick-up, in vitro fertilization (IVF) or intra-cytoplasmic sperm injection, with embryo transfer or vitrification on day 5 of culture, as described by De Croo et al. [17].

The height and weight of the patients were used to calculate the body mass index (BMI). A BMI limit was applied within the cohort, such that patients with a score exceeding 35.0 kg/m^2^ were consulted to lose weight before starting stimulation for fertility treatment. The patients were distributed to three BMI groups according to the criterion of the World Health Organization [18] as follows: normal weight group (18.5 ≤ BMI ≤ 24.9 kg/m^2^), overweight (25.0 ≤ BMI ≤ 29.9 kg/m^2^), and obese group (BMI ≥ 30.0 kg/m^2^).

In the current study, every patient seeking assisted reproductive services at the Ghent University Hospital (Ghent, Belgium) and willing to participate in the study was retained in order to exclude any sampling bias. To determine the required sample size to study the hypothesis, a power analysis was performed. Based on historical data of the Ghent University Hospital, a standard cohort of patients searching for ART is expected to have about 5 times more normal weight patients as compared with obese patients. Accordingly, a population size analysis was performed with the G*Power 3.1 software with an alpha risk less than 0.05, a power greater than 0.80 and an allocation ratio obese patients/normal weight patients of 0.2 (t-test, difference between two independent means). Relying on the results reported by Valckx et al. [14], a sample size effect of 0.614 was estimated, based on reproductive outcome data (2PN percentages) of the normal weight vs the obese group. Accordingly, a minimum group size of 20 in the obese group and 100 patients in the normal weight group was calculated. Unfortunately, this number was not reached as for logistic and budgetary reasons, the sampling was ceased after a two-years sampling period with 93 normal weight patients and 17 obese patients, which was calculated to allow to reach an alpha risk less than 0.070 and a power greater than 0.8 (G*Power 3.1 software). Moreover, also for other parameters, e.g. for some of the most relevant FA in FF (i.e. C18:1n-9, n-3 and n-6 long-chain FA in the phospholipid fraction), a power analysis was performed. The number of samples required within each BMI-group generally was lower and never exceeded the sample size calculated from the power analysis based on the 2PN/oocytes.

### Grouping of patients

From the group of 93 normal weight patients, 9 had to be discarded for various reasons (too low semen quality (*n* = 4), unintended repeated registration of the same patient during the 2-years collection period (*n* = 3) and unreliable fatty acid results (*n* = 2)).

Accordingly, the normal weight group, overweight and obese group included 84, 37 and 17 patients. In addition, patients were equally classified into three age-related groups irrespective of BMI: 20–30 years (*n* = 50), 31–34 years (*n* = 43), and ≥ 35 years (*n* = 45), respectively.

### Scoring oocyte, cleavage stage embryos and blastocysts

The number of retrieved and injected oocytes as well as the number of normal fertilized oocytes, which are zygotes showing 2 pronuclei (2PN), were recorded during the ART process. Additional parameters, monitored in the current study, included the number and proportion of embryos with 6 and 8 cells or more on day 3 as well as the number and proportion of embryos developing between days 3 and 5 (ED3-5) and the number and proportion of expanded blastocysts on day 5 (EB5). Expanded blastocysts are embryos that show a fluid cavity with an inner cell mass and trophectoderm layer on day 5 (at minimum a score of 3CC according to Gardner and Schoolcraft, 1999) [19]. Furthermore, the embryo utilization rate (EUR) was calculated as the proportion of 2PN that led to transferred and cryopreserved embryos. One patient with fertilization failure (without 2PN) was excluded from the related analysis of ED3-5/2PN, EB5/2PN and EUR. Lastly, the cumulative live birth rate was calculated from the number of live births up to two years after oocyte pick-up, of one patient from the normal weight group information on the live birth outcome was missing. Accordingly, this patient was excluded from cumulative birth rate calculation.

### Sampling and analysis of FF

At the time of oocyte retrieval, the pooled FF from multiple follicles between the sizes of 14 and 24 mm were collected from patients, followed by centrifugation at 1500 × g for 10 min, after which the supernatant was stored at -80 °C in the biobank of the Ghent University Hospital under the operational management of Bioresource UZ Ghent (ID: BE 71,067,049) until the analysis of TFA composition and FA composition in different lipid fractions.

In terms of TFA analysis, fatty acid methyl esters (FAME) were prepared from 0.2 mL of FF samples using a direct transesterification procedure described by Vlaeminck et al. [20], with minor modifications. Briefly, toluene (1 mL) containing the internal standard (non-esterified C21:0; Sigma Aldrich, Diegem, Belgium) and methanolic NaOH (2 mL; 0.5 M) were added and the mixture was incubated at 70 °C for 60 min. This was followed by 30 min of incubation at 50 °C after the addition of methanolic HCl (3 mL), which was prepared by dissolving acetyl chloride in methanol (5/1, vol/vol). The FAME were extracted with hexane.

Prior to FA measurements in different lipid fractions (free FA, cholesterol esters, phospholipids, triglycerides), total FF lipids were extracted following the protocol in our previous study [21] and the extract was separated using the SPE column method and then methylated according to Valckx et al. [14].

The analysis of FAMEs was carried out with a gas chromatograph (HP7890A, Agilent Technologies, Diegem, Belgium) equipped with a SP-2560 capillary column (75 m × 0.18 mm × 0.14 µm film thickness; Supelco, Bellefonte, PA, USA) and a flame ionization detector. The temperature program was as follows: initially 70 °C, increasing by 50 °C/min to 175 °C and holding for 13 min, followed by a second increase at 5 °C/min to 215 °C, which was held for 20 min. The inlet and detector temperatures were 250 and 255 °C, respectively. The split ratio was 25:1. The flow rate of the hydrogen carrier gas was 1 mL/min. Identities of FA peaks were determined using mixtures of methyl ester standards (GLC463, Nu-chek prep, MN, USA). Additionally, 4 fatty acids were identified which were not included in the GLC463, i.e. C20:1n-7, C16:1n-9, C20:4n-3 and C22:5n-6. C20:1n-7 was identified based on its relative retention time compared with the position of C20:1n-9. C16:1n-9, C20:4n-3 and C22:5n-6 were identified based on reference milk and fish oil samples in our lab. Identification of fatty acids in these samples have been done in detail using a prior separation by silver thin layer chromatography and identification (if necessary) by GC–MS of DMOX-derivates in previous studies. Individual fatty acid proportions were expressed as grams per 100 g of the FAME. Total FAME were calculated based on both identified and unidentified peaks appearing between C12:0 and C22:6n-3. Quantification of FAME was based on the conversion of peak areas to the mass proportion of FA by a theoretical response factor for each FA [22, 23]. For the unidentified peaks, a response factor of 1 was assumed.

Analytical accuracy was verified by determination of inter-assay coefficients of variance of 9 samples analyzed on 2 different days.

### Statistical analysis

All statistical analyses were performed with SPSS software version 20.0. The data were tested for normality using Shapiro–Wilk (*n* < 50) and Kolmogorov–Smirnov (n ≥ 50) tests. Non-normally distributed variables were log-transformed before statistical analysis. Data of some variables were not normally distributed, even after log-transformation. In these cases, the original data were subjected to non-parametric statistical analysis. Assisted reproductive technology outcome and TFA composition of FF were compared across BMI (normal weight, overweight, and obese group) and age (20–30, 31–34, and 35–42 years) categories using a one-way ANOVA or non-parametric Kruskal–Wallis tests, except for the cumulative live birth rates in the three BMI and in the three age groups, which were compared through a Fisher’s exact tests. The BMI, age, specific FA and FA groups in the FF of the first quartile vs. the fourth quartile of ED3-5/2PN, EB5/2PN, and EUR were compared by an independent t-test or a non-parametric Mann–Whitney U test. The correlations between the outcome of ED3-5/2PN, EB5/2PN, EUR and specific FA as well as FA groups were investigated within BMI and age groups by Pearson's correlation analysis. The fatty acids or fatty acid groups, which showed differences between Q1 and Q4 and/or significantly associated with ED3-5/2PN, EB5/2PN or EUR, were further checked for mutual correlations by Pearson's correlation analysis. Mutually uncorrelated (|*r*|< 0.6) were included in a multiple linear regression analysis with ED3-5/2PN, EB5/2PN, and EUR as independent variables. Statistical significance was set at *P* < 0.05 and trend at 0.05 ≤ *P* < 0.10.

## Results

### Patient characteristics and ART outcome in relation to BMI

According to the classification by BMI, 60.9% patients (*n* = 84) of normal weight, 26.8% patients (*n* = 37) who were overweight and 12.3% (*n* = 17) obese women. Body mass index did not affect ART laboratory parameters, except for the proportion of embryos with ≥ 8 cells on day 3, which was significantly greater (*P* < 0.05) in the obese group than in the overweight and normal weight groups (Table 1).

**Table 1:** Patient characteristics and clinical ART outcome among normal weight, overweight and obese groups

**Parameters**	**Statistics**^a^	**Normal weight**	**Overweight**	**Obese**	***P*-value**
		**18.5 ≤ BMI ≤ 24.9**	**25.0 ≤ BMI ≤ 29.9**	**BMI ≥ 30.0**	
Patient, n	/	84	37	17	
Body mass index, kg/m^b^	K	**21.8**^**a**^** (20.6–23.3)**	**26.5**^**b**^** (25.7–28.4)**	**32.6**^**c**^** (31.3–34.7)**	< 0.001
Age, year	K	32.0 (30.0–36.8)	32.0 (29.0–35.0)	31.0 (28.0–34.5)	0.343
Total doses of urinary gonadotropins, IU	K	2252 (1819–3094)	2626 (1950–3601)	3019 (2072–4538)	0.124
Total doses of recombinant gonadotropins, IU	K	2363 (1351–3376)	1575 (1369–2104)	2288 (1425–2441)	0.414
Oocytes retrieved, n	K	14.0 (10.3–18.0)	13.0 (10.0–17.0)	13.0 (8.50–17.0)	0.610
Oocytes injected + oocytes inseminated, n	K	12.0 (9.00–15.0)	11.0 (9.00–14.0)	10.0 (7.50–14.0)	0.394
Two-pronuclei oocytes (2PN)^b^, n	K	7.00 (5.00–10.0)	8.00 (6.00–9.00)	7.00 (4.50–10.0)	0.875
2PN / (oocytes injected + oocytes inseminated), %	K	71.4 (50.0–80.0)	77.4 (64.6–87.5)	66.7 (58.6–83.3)	0.218
Embryos with ≥ 6 cells on day 3, n	K	5.00 (3.00–8.00)	6.00 (5.00–8.50)	5.00 (4.00–9.00)	0.332
Embryos with ≥ 6 cells on day 3 / 2PN, %	K	80.0 (60.0–93.3)	84.6 (71.4–100)	90.0 (69.0–100)	0.079
Embryos with 8 cells on day 3, n	K	3.00 (1.00–4.00)	4.00 (2.00–5.00)	4.00 (2.00–6.00)	0.093
Embryos with 8 cells on day 3 / 2PN, %	A	**37.2**^a^ **(32.2–42.3)**	**44.7**^a^ **(36.6–52.9)**	**59.5**^b^ **(48.6–70.5)**	0.002
Embryo development between days 3 and 5 (ED3-5)	K	4.00 (2.00–6.00)	4.00 (2.00–6.00)	4.00 (2.50–6.50)	0.794
ED3-5 / 2PN, %	A	54.7 (48.5–60.8)	59.5 (49.5–69.5)	65.4 (53.0–77.7)	0.309
Expanded blastocysts on day 5 (EB5), n	K	1.00 (0.00–3.00)	3.00 (0.00–5.00)	2.00 (0.50–4.50)	0.520
EB5 / 2PN, %	K	20.0 (0.00–45.5)	33.3 (0.00–56.3)	30.0 (7.14–50.0)	0.577
Embryo utilization rate (EUR)^c^, %	K	40.0 (22.2–53.3)	43.8 (14.3–69.0)	50.0 (31.0–70.8)	0.194
Cumulative live birth rate^d^, %	Fisher's exact	50.6	45.9	47.1	0.873

Mean (95% CI), in case of normal data distribution or Median (25–75%), in case of non-normal data distribution, are presented. Parameters which show significant differences (*P* < 0.05) are presented in bold

^a^Statistics: A (ANOVA test): ANOVA followed by Least Significant Difference test to determine significance between BMI groups at *P* < 0.05, which are indicated by superscripted letters (a, b); K (Kruskal Wallis test): Kruskal Wallis test followed by a post-hoc Mann–Whitney-Wilcoxon test to determine significance between BMI groups at *P* < 0.05, which are indicated by superscript letters (a, b, c)

^b^From intra-cytoplasmic sperm injection and in vitro fertilization

^c^(embryos transferred + number of cryopreserved embryos) / 2PN

^d^Live birth per cycle, expressed per 100 cycle attempts

### Patient characteristics and ART outcome in relation to age

Women aged ≥ 35 years had less expanded blastocysts on day 5 (both in absolute numbers as well as proportionally to 2PN (EB5/2PN)) than women between 20 and 30 years, which was also reflected in the lower proportion of embryos developing between days 3 and 5 (ED3-5/2PN) and the lower embryo utilization rate (EUR) (Table 2).

**Table 2:** Patient characteristics and clinical ART outcome among young, middle and old groups

**Parameters**	**Statistics**^a^	**Young**	**Middle**	**Old**	***P*-value**
		**20–30 years**	**31–34 years**	**35–42 years**	
Patient, n	/	50	43	45	/
Body mass index, kg/m^b^	K	24.2 (21.7–27.0)	23.3 (21.0–26.9)	23.6 (21.4–25.8)	0.612
Age, year	K	**29.0**^**a**^** (27.0–30.0)**	**32.0**^**b**^** (31.0–33.0)**	**37.0**^**c**^** (36.0–39.0)**	< 0.001
Total doses of urinary gonadotropins, IU	K	**2026**^**a**^** (1614–2851)**	**2251**^**ab**^** (1950–2691)**	**3186**^**b**^** (2250–3901)**	0.003
Total doses of recombinant gonadotropins, IU	A	**1815**^**a**^** (1373–2257)**	**2224**^**a**^** (1555–2892)**	**3175**^**b**^** (2353–3997)**	0.011
Oocytes retrieved, n	K	14.0 (10.0–18.0)	14.0 (10.0–18.0)	13.0 (9.50–16.5)	0.460
Oocytes injected + oocytes inseminated, n	K	10.5 (8.00–14.0)	12.0 (9.00–16.0)	11.0 (8.50–14.0)	0.466
Two-pronuclei oocytes (2PN)^b^, n	K	7.00 (4.75–10.0)	8.00 (5.00–12.0)	7.00 (5.00–9.00)	0.456
2PN / (oocytes injected + oocytes inseminated), %	K	73.2 (50.0–81.8)	71.4 (55.6–87.5)	71.4 (57.3–81.8)	0.979
Embryos with ≥ 6 cells on day 3, n	K	6.00 (3.00–8.00)	5.00 (3.00–9.00)	5.00 (3.00–8.00)	0.933
Embryos with ≥ 6 cells on day 3 / 2PN, %	K	85.7 (70.3–100)	71.4 (62.5–100)	80.0 (60.0–91.4)	0.119
Embryos with 8 cells on day 3, n	K	3.00 (1.00–4.25)	4.00 (2.00–5.00)	3.00 (1.00–4.50)	0.603
Embryos with 8 cells on day 3 / 2PN, %	A	43.3 (36.4–50.2)	44.8 (36.7–52.9)	38.0 (31.2–44.7)	0.377
Embryo development between days 3 and 5 (ED3-5), n	K	4.00 (3.00–6.00)	4.00 (2.00–7.00)	3.00 (2.00–5.00)	0.266
ED3-5 / 2PN, %	K	**66.7**^**a**^** (50.0–81.7)**	**62.5**^**ab**^** (37.5–77.8)**	**46.2**^**b**^** (28.6–70.0)**	0.009
Expanded blastocysts on day 5 (EB5), n	K	**3.00**^**y**^** (0.00–4.00)**	**2.00**^**yz**^** (0.00–5.00)**	**1.00**^**z**^** (0.00–3.00)**	0.097
EB5 / 2PN, %	K	**40.0**^**a**^** (0.00–51.7)**	**28.6**^**ab**^** (0.00–45.5)**	**13.3**^**b**^** (0.00–32.1)**	0.028
Embryo utilization rate (EUR)^c^, %	K	**50.0**^**a**^** (30.4–67.9)**	**42.9**^**ab**^** (20.0–61.5)**	**33.3**^**b**^** (19.1–44.4)**	0.020
Cumulative live birth rate^d^, %	Fisher's exact	57.1	46.5	42.2	0.480

Mean (95% CI), in case of normal data distribution or Median (25%-75%), in case of non-normal data distribution, are presented. Parameters which show significant differences (*P* < 0.05) or a trend (0.05 ≤ *P* < 0.10) are presented in bold

^a^Statistics: A (ANOVA test): ANOVA followed by Least Significant Difference test to determine significance between age groups at *P* < 0.05, which are indicated by superscripted letters (a, b). K (Kruskal Wallis test): Kruskal Wallis test followed by a post-hoc Mann–Whitney-Wilcoxon test to determine trends (0.05 ≤ *P* < 0.10) or significance (*P* < 0.05) between age groups, which are indicated by superscripted letters (y, z and a, b, c respectively)

^b^From in vitro fertilization or intra-cytoplasmic sperm injection

^c^(embryos transferred + number of cryopreserved embryos) / 2PN

^d^Live birth per cycle, expressed per 100 cycle attempts

### Follicular fluid FA in relation to embryo outcome based on first vs. fourth quartile classification

To assess relationships between FF FA and ART outcome, data of ED3-5/2PN, EB5/2PN, and EUR were split into quartiles. The two extreme quartiles were compared (Table 3). Greater proportions of C20:5n-3, C22:6n-3, and total n-3 PUFA, and a lower ratio of n-6 to n-3 PUFA were found (*P* < 0.05) in the FF of the first compared with the fourth quartile, with the latter being considered the quartile with the best ART outcome (Table 3). Furthermore, we noted that the patients of the fourth quartile were relatively younger than patients of the first quartile (*P* < 0.10) (Table 3). Negative associations of n-3 PUFA with embryo outcome were reflected in each lipid fraction. Nevertheless, changes in FF cholesterol esters and free FA fractions were relatively minor, while higher proportions of C20:5n-3, C22:6n-3 and total n-3 PUFA were observed in FF phospholipids and triglycerides of the Q1 group (Supplementary Table 1). Furthermore, higher proportions of C16:0 were found in FF triglycerides of the Q4 group as compared with the Q1 group (Supplementary Table 1).

**Table 3:** Comparison of BMI, age and proportions of specific FF FA and FA groups of the least (1st quartile, Q1) and best (4th quartile, Q4) performing quartiles, based on the assisted reproductive technology outcome parameters related to embryos developing between days 3 and 5 relative to oocytes with two pronuclei (ED3-5/2PN), expanded blastocysts on day 5 relative to oocytes with two pronuclei (EB5/2PN) and embryo utilization rate (EUR)

Item	ED3-5/2PN				EB5/2PN				EUR			
	**Q1**	**Q4**	**Statistics**^a^	***P*-value**	**Q1**	**Q4**	**Statistics**^**1**^	***P*-value**	**Q1**	**Q4**	**Statistics**^**1**^	***P*-value**
Proportion, %	≤ 38.8	≥ 77.4			= 0.00	≥ 48.5			≤ 21.8	≥ 59.2		
Patient, n	34	34			39	32			34	34		
Body mass index, kg/m^2^	23.6	25.6	MW	0.115	24.0	24.3	MW	0.885	24.2	25.7	MW	0.136
Age, year	**34.4**	**32.3**	T	**0.046**	**33.0**	**30.0**	MW	**0.056**	**32.8**	**31.0**	T	**0.048**
Fatty acid (FA), % by weight												
C16:0	20.9	21.0	T	0.729	20.7	21.0	T	0.471	20.7	20.9	T	0.579
C16:1n-7	1.13	1.17	T-log	0.674	1.10	1.12	T-log	0.865	1.11	1.14	T-log	0.751
C18:0	8.23	8.15	T-log	0.645	8.25	8.04	MW	0.262	8.31	8.15	T-log	0.402
C18:1n-9	16.1	16.7	T	0.214	16.4	16.6	T	0.546	16.2	16.6	T	0.384
C18:2n-6	23.9	24.1	T	0.753	23.8	24.4	T	0.396	24.3	23.7	T	0.503
C18:3n-3	0.37	0.38	MW	0.524	0.36	0.40	T-log	0.108	0.37	0.40	T-log	0.185
C20:4n-6	7.91	8.20	T	0.400	7.95	8.36	T	0.209	**7.93**	**8.54**	T	**0.062**
C20:5n-3	**0.74**	**0.51**	T-log	**0.006**	**0.59**	**0.50**	MW	**0.030**	**0.66**	**0.51**	T-log	**0.022**
C22:6n-3	**2.70**	**2.19**	T	**0.002**	**2.48**	**2.15**	T-log	**0.003**	**2.50**	**2.22**	T-log	**0.037**
Saturated FA	31.4	31.3	T	0.772	31.3	31.2	T	0.777	31.3	31.3	T	0.839
Monounsaturated FA	21.3	21.5	T	0.700	21.4	21.3	MW	0.954	21.2	21.4	T	0.731
* n*-3 Polyunsaturated FA	**4.65**	**3.82**	T-log	**0.001**	**4.35**	**3.81**	T-log	**0.007**	**4.42**	**3.92**	T-log	**0.019**
* n*-6 Polyunsaturated FA	34.8	35.4	T	0.318	34.8	35.8	T	0.145	35.2	35.3	T	0.874
* n*-6: *n*-3 Polyunsaturated FA	**7.45**	**9.25**	T-log	**0.001**	**8.18**	**9.52**	T	**0.002**	**7.95**	**8.98**	T-log	**0.021**

Mean, in case of normal data distribution or Median, in case of non-normal data distribution, are presented. Parameters which show significant differences (*P* < 0.05) or a trend (0.05 ≤ *P* < 0.10) are presented in bold

^a^Statistics: *MW* Mann–Whitney U tests, *T* Independent T-test, *T-log* a T-test has been performed when log-transformed (T-log) data were normally distributed. For log-transformed data (T-log), log-back transformations were carried out to calculate the geometric mean

Sum of saturated fatty acids (Saturated FA) = Σ (C12:0, C13:0, C14:0, C15:0, C16:0, C17:0, C18:0, C20:0, C22:0, C24:0)

Sum of Monounsaturated fatty acids (Monounsaturated FA) = Σ (C14:1n-5, C16:1n-9, C16:1n-7, C18:1n-9, C18:1n-7, C20:1n-7, C20:1n-9, C22:1n-9, C24:1n-9)

Sum of n-3 Polyunsaturated fatty acids (n-3 Polyunsaturated FA) = Σ (C18:3n-3, C20:3n-3, C20:4n-3, C20:5n-3, C22:5n-3, C22:6n-3)

Sum of n-6 Polyunsaturated fatty acids (n-6 Polyunsaturated FA) = Σ (C18:2n-6, C18:3n-6, C20:2n-6, C20:3n-6, C20:4n-6, C22:4n-6, C22:5n-6)

### Associations of BMI, Age, and FA in FF with embryo outcome

Within BMI and age groups, we found significant correlations between the proportions of C18:0, C18:1n-9, C20:4n-6, C20:5n-3 and monounsaturated FA (MUFA) in FF and the performance of embryo development (Additional Files 1 and File 2). Except for the high correlation between the proportion of C18:1n-9 and MUFA (*r* = 0.826), other mutual correlations were weak (*r* < 0.30). Then, the correlation analysis was further performed for the proportions of FF C20:5n-3, C22:6n-3, total n-3 PUFA, and the ratio of n-6 to n-3 PUFA which showed differences between Q1 and Q4; their mutual correlations were strong (all *r* > 0.60). Moreover, the proportions of several FA in FF differed between BMI and age groups (Supplementary Tables 2 and 3). Therefore, the proportions of FF C18:0, C18:1n-9, C20:4n-6, C20:5n-3, BMI and age as well as TFA concentration were included in regression models on embryo outcome. Within the entire cohort (Table 4), the proportion of C20:5n-3 was negatively correlated with ED3-5/2PN (β = -0.21, *P* = 0.023) and EUR (β = -0.17, *P* = 0.072), whereas EB5/2PN tended to be negatively correlated with age (β = -0.16, *P* = 0.063) and positively with the TFA concentration in FF (β = 0.24, *P* = 0.010). A trend was also found for the association of the proportion of C20:4n-6 in FF with EUR (β = 0.17, *P* = 0.054). When patients were further categorized into normal weight and overweight/obese groups, the regression results in the normal weight group were similar to those of the whole cohort, embryo development parameters correlated negatively with age (*P* < 0.10; tendency) or with the proportion of C20:5n-3 (*P* < 0.05) in FF (Table 4). Within the overweight/obese group, regression models also indicated that C20:5n-3 was negatively associated with ED3-5/2PN (β = -0.34, *P* = 0.043), but various other FF FA were associated with embryo parameters: C18:1 n-9 (β = 0.35, *P* = 0.016) was associated with ED3-5/2PN, whereas the proportion of C18:0 (β = -0.34, *P* = 0.018) and C20:4 n-6 (β = 0.26, *P* = 0.086) were associated with EUR (Table 4). However, none of the multiple regressions explained more than 23% of the variation in the dataset.

**Table 4:** Standardised regression coefficients relating BMI, age and total and specific FF FA to embryo outcome

Groups	Embryo outcome	R Square	Variables	BMI (kg/m^2^)	Age (years)	TFA (µM)	C18:0 (g/100 g FA)	C18:1n-9 (g/100 g FA)	C20:4n-6 (g/100 g FA)	C20:5n-3 (g/100 g FA)
**All patients (*n* = 137)**	ED3-5/2PN	0.14	Partial R Square	0.00	0.02	0.01	0.00	0.00	0.01	**0.04**
			β	0.01	-0.14	0.12	0.05	0.05	0.09	**-0.21**
			*P*-value	0.879	0.102	0.166	0.559	0.518	0.285	**0.023**
	EB5/2PN	0.15	Partial R Square	0.01	**0.02**	**0.05**	0.00	0.00	0.01	0.02
			β	-0.08	**-0.16**	**0.24**	0.05	0.02	0.12	-0.15
			*P*-value	0.376	**0.063**	**0.010**	0.602	0.844	0.168	0.108
	EUR	0.12	Partial R Square	0.00	0.01	0.00	0.00	0.00	**0.03**	**0.02**
			β	0.07	-0.10	0.07	-0.05	0.02	**0.17**	**-0.17**
			*P*-value	0.456	0.243	0.433	0.556	0.771	**0.054**	**0.072**
**Normal weight (*n* = 83)**	ED3-5/2PN	0.17	Partial R Square		**0.04**	**0.03**	0.02	0.02	0.01	**0.05**
			β		**-0.21**	**0.19**	0.15	-0.13	0.07	**-0.23**
			*P*-value		**0.071**	**0.091**	0.182	0.235	0.508	**0.046**
	EB5/2PN	0.14	Partial R Square		**0.04**	0.02	0.00	0.00	0.02	0.01
			β		**-0.20**	0.16	0.01	-0.05	0.16	-0.13
			*P*-value		**0.082**	0.172	0.954	0.667	0.148	0.277
	EUR	0.19	Partial R Square		0.03	0.01	0.02	0.03	0.03	**0.05**
			β		-0.18	0.11	0.14	-0.16	0.18	**-0.24**
			*P*-value		0.109	0.303	0.215	0.130	0.107	**0.040**
**Overweight + obese (*n* = 54)**	ED3-5/2PN	0.23	Partial R Square		0.00	0.00	0.01	**0.10**	0.04	**0.07**
			β		-0.07	-0.07	-0.11	**0.35**	0.23	**-0.34**
			*P*-value		0.606	0.643	0.445	**0.016**	0.116	**0.043**
	EB5/2PN	0.19	Partial R Square		0.00	**0.06**	0.01	0.00	0.00	0.02
			β		-0.06	**0.30**	0.11	0.05	0.06	-0.18
			*P*-value		0.704	**0.062**	0.439	0.708	0.690	0.285
	EUR	0.21	Partial R Square		0.00	0.00	**0.10**	0.04	**0.05**	0.03
			β		-0.01	-0.00	**-0.34**	0.22	**0.26**	-0.20
			*P*-value		0.948	0.982	**0.018**	0.119	**0.086**	0.231

Significant (*P* < 0.05) regression coefficients or coefficients tending to be significantly different from zero (0.05 ≤ *P* < 0.10) are presented in bold

## Discussion

Morphological criteria commonly used to select embryos at the cleavage stage are insufficient to predict their developmental potential [11], whereas transfer at blastocyst rather than cleavage stage is associated with greater clinical pregnancy rates per embryo transfer [12]. Hence, in the current study, embryo development between days 3 and 5 and the expansion at the blastocyst stage on day 5 have been particularly emphasized to assess their relations with BMI, age, and FF FA concentration and composition.

More precisely, the embryo development between days 3 and 5 and the number of expanded blastocysts on day 5 did not differ between BMI groups. In contrast, Leary et al. [16] demonstrated that oocytes from women with a BMI exceeding 25 kg/m^2^ were less likely to complete development post-fertilization, and blastocysts contained fewer cells, notably in the trophectoderm. In addition, García-Ferreyra et al. [24] and Comstock et al. [25] reported that the blastocyst formation rate was significantly better in a normal-weight group than in overweight and obese groups. Moreover, Esteves et al. [26] demonstrated that the probability of a mature oocyte turning into an euploid blastocyst decreased progressively with female age. Similarly, in our study, embryo development between days 3 and 5 and blastocyst quality on day 5 were enhanced in the age-group between 20 and 30 years compared with the group aged over 35 years. Furthermore, in our study, females of the obese group were younger than women of normal weight on average, while other BMI studies showed the opposite to be true [24, 25], with relatively younger patients in the obese vs. the normal weight groups, although the age-distribution did not differ significantly between weight groups in these studies. Accordingly, age effect could have overruled the adverse effects of obesity on embryo development in our study, which could explain the inconsistent results between our results and those of the previous BMI studies.

Variation in FA concentration and composition in the follicular microenvironment was suggested to be one of the potential factors explaining BMI-related impact on the reproductive outcome [14]. However, in the current study, alterations in FF FA concentration and composition related to blastocyst development and quality irrespective of BMI; i.e., the good blastocyst group (4^th^ quartile) had lower proportions of n-3 PUFA, C20:5n-3 and C22:6n-3 in FF as compared with the poor blastocyst group (1^st^ quartile). In line with our findings, Ruiz-Sanzs et al. [13] showed that total n-3 PUFA, particularly C22:6n-3, in FF of large follicles correlated negatively with top-quality embryos and fertilization rate. Additionally, the study by Zarezadeh et al. [27] indicated that C18:3n-3, C20:5n-3 and total n-3 PUFA in the FF phospholipid fraction inversely correlated with the number of embryos. Although FF provides the micro-environment from which the oocytes retrieve FA and other nutrients, only very few studies reported oocyte FA for obvious ethical and technical reasons. Indeed, for ethical reasons, only unfertilized and discarded human oocytes [28, 29] would be available for such studies. From a technical point of view, Matorras et al. [29] indicated the need to analyse pooled oocytes, as individual oocyte lipid mass was well below the sensitivity of the analytical method. Given the particular importance of PUFA, including n-3 PUFA, in membrane fluidity and integrity [30], it could be speculated that a certain proportion of n-3 PUFA is required in membrane phospholipids to guarantee optimal biological functionality. As such, it could be further speculated that oocytes of better quality were capable to extract more n-3 PUFA from their surrounding environment, i.e. the FF, which could result in lower proportions of residual n-3 PUFA in FF. However, n-3 PUFA were present in very small proportions in oocyte phospholipids of ewes either or not supplemented with fish oil, while C20:5n-3 and C22:6n-3 even were virtually absent [31]. Although these n-3 PUFA were detected in human oocytes, their proportion never exceeded 1% of the total oocyte FA [29]. Moreover, comparison of the FF FA proportions of our study with the FA composition in oocytes reported by Matorras et al. [29] suggests an enrichment of MUFA in lipids of the oocytes (> 50%; proportion of TFA) as compared with the FF lipids (< 25%). This suggests, oocytes to preferentially take up MUFA from the follicular environment rather than n-3 PUFA. Hence, the presumably conflicting associations between n-3 PUFA and fertility outcome, depending on the biological matrix in which n-3 PUFA is assessed needs further investigation. It further should be noted that the formerly discussed associations are based on FA proportions. Although proportions of FF n-3 PUFA and specifically C20:5n-3 and C22:6n-3 differed between good and poor embryo groups in the current study, their concentrations were similar in both groups of our cohort. Additionally, other factors affecting fertility may be correlated with n-3 PUFA proportions, which may complicate interpretation. E.g. within our cohort, age and FF n-3 PUFA proportions were weakly but positively related (*r* = 0.234), which had also been reported previously by Ruiz-Sanzs et al. [13]*.*

Phospholipid and triglyceride fractions showed similar differences in FA proportions between the Q1 and Q4 groups as observed in TFA, while relatively minor differences were observed in the free fatty acid and cholesterol-ester fractions. As almost half of the TFA are included in the phospholipid fraction [14], consistent changes in TFA and phospholipid FA as well as similar associations with embryo outcome are not surprising and have been reported before [27]. On the other hand, studies on the association between FF FA in the triglyceride fraction and embryo outcome is very limited. The only report by Zarezadeh et al. [27] was inconsistent with our results. Here, the percentages of C16:0 in FF triglyceride seemed to be positively associated with embryo development. However, it should be noted that the proportions of C16:0 in FF triglyceride were positively correlated with the patients’ BMI in our study.

Further, the normal and overweight/obese patients were separated. Within the group of normal weight patients, the formerly mentioned negative relationships between FF C20:5n-3 and the proportion of embryos developing between days 3 and 5 or the embryo utilization rate were confirmed. Additionally, the proportion of embryos developing between days 3 and 5 and expanded blastocyst on day 5 tended to negatively correlate with age within this group. However, only the proportion of embryos developing between days 3 and 5 negatively related to C20:5n-3 within the group of overweight/obese women, while age was not associated with any of the ART outcome parameters. This lack of association with age should be taken with some caution given the over-representation of younger women in the overweight/obese group. Indeed, age was less equally distributed within the overweight/obese group (20–30 years, 42.6%; 31–34 years, 27.8%; ≥ 35 years, 29.6%) than in the normal weight group (31.3, 33.7, and 34.9%, respectively). Another interesting finding is that the proportion of C18:1n-9 was positively correlated with embryo development while C18:0 was negatively correlated with blastocyst development in the overweight/obese group. Similarly, Mirabi et al. [32] reported that the concentration of C18:1n-9 in serum was positively associated with the number of mature oocytes while the concentration of C18:0 in FF were negatively correlated with metaphase II oocytes. Furthermore, in agreement with our findings, previous studies in dairy cow models have demonstrated that the addition of C18:0 during oocyte maturation had negative effects on maturation, fertilization, and cleavage rate as well as blastocyst yield [33], while C18:1n-9 could counteract these adverse effects [34].

Finally, we must acknowledge potential limitations of the current findings: although BMI, age and some FF FA have been related to embryo quality, the parameters monitored in the current study only explained a limited proportion of the variation in embryo development stage between days 3 and 5, and blastocyst quality on day 5. Obviously, also other factors, which have not been monitored here might affect embryo quality and reproductive outcome, e.g. including, eating behaviour, unhealthier lifestyle (e.g., alcohol consumption and smoking) and other health behavioural habits (e.g., exercise and sleeping pattern).

## Conclusion

This study reports the relationships between female age, FF FA composition, and embryo outcome. The proportions of n-3 PUFA, particularly C20:5n-3 and C22:6n-3 were reduced in the quartile of patients with the best embryo outcome of the current cohort. This group of patients was also younger. However, the embryo quality parameters of overweight-obese patients were not associated with age but were positively associated with FF C18:1n-9 and negatively with the proportions of C18:0 or C20:5n-3.

## Supplementary Information

**Additional file 1.** Correlation analysis for proportions (% by weight) of specific FF FA and FA groups with embryo outcome among normal weight women (n = 83) in 3 age groups.

**Additional file 2.** Correlation analysis for proportions (% by weight) of specific FF FA and FA groups with embryo outcome among overweight/obese women (n = 54) in 3 age groups.

**Additional file 3:** **Supplementary 1.** Comparison of the proportions of specific FF FA and FA groups in free fatty acids (FFA), phospholipids (PL), cholesterol esters (CHE) and triglycerides (TG)) of the least (1st quartile, Q1) and best (4th quartile, Q4) performing quartiles, based on the assisted reproductive technology outcome parameters related to embryos developing between days 3 and 5 relative to oocytes with two pronuclei (ED3-5/2PN), expanded blastocysts on day 5 relative to oocytes with two pronuclei (EB5/2PN) and embryo utilization rate (EUR). Means or Median of parameters which show significant differences (P < 0.05) or a trend (0.05 ≤ P < 0.10) are presented in bold. **Supplementary Table 2.** Concentration of TFA or FA families and relative proportions of individual FF FA of normal weight, overweight and obese women. **Supplementary Table 3.** Concentration of TFA or FA families and relative proportions of individual FF FA in three age groups.

## Data Availability

The datasets used and/or analysed during the current study are available from the corresponding author on reasonable request.

## Abbreviations

FA: Fatty acid
FF: Follicular fluid
BMI: Body mass index
ART: Assisted reproductive technology
Q4: 4^Th^ quartile
Q1: 1^St^ quartile
ED3-5: Embryos developing between days 3 and 5
EB5: Expanded blastocysts on day 5
2PN: Two pronuclei
ED3-5/2PN: The proportion of embryos developing between days 3 and 5 to the number of oocytes with two pronuclei
EB5/2PN: The proportion of expanded blastocysts on day 5 to the number of oocytes with two pronuclei
MUFA: Monounsaturated fatty acid
PUFA: Polyunsaturated fatty acid
EUR: Embryo utilization rate
TFA: Total fatty acid
IVF: In vitro fertilization
