# Supplementary material for: The fatty acid composition in follicles is related to the developmental potential of oocytes up to the blastocyst stage: a single-centre cohort study
Source: Reprod Biol Endocrinol. 2022 Jul 25;20:107. doi: 10.1186/s12958-022-00974-7 (PMC9310456; doi:10.1186/s12958-022-00974-7)
Supplement: Supplementary file 1 — Additional file 1. Correlation analysis for proportions (% by weight) of specific FF FA and FA groups with embryo outcome among normal weight women (n = 83) in 3 age groups. [file 12958_2022_974_MOESM1_ESM.docx]

**Additional File 1. Correlation analysis for proportions (% by weight) of specific FF FA and FA groups with embryo outcome among normal weight women**

**(n = 83) in 3 age groups.**

| **Items** | **20-30 years** | | | | | | **31-34 years** | | | | | | **≥ 35 years** | | | | | |
| --- | --- | --- | --- | --- | --- | --- | --- | --- | --- | --- | --- | --- | --- | --- | --- | --- | --- | --- |
|  | **ED3-5/2PN** | | **EB5/2PN** | | **EUR** | | **ED3-5/2PN** | | **EB5/2PN** | | **EUR** | | **ED3-5/2PN** | | **EB5/2PN** | | **EUR** | |
| **Fatty acid (FA)** | r | *P*-value | r | *P*-value | r | *P*-value | r | *P*-value | r | *P*-value | r | *P*-value | r | *P*-value | r | *P*-value | r | *P*-value |
| C16:0 | 0.320 | 0.111 | 0.208 | 0.308 | 0.292 | 0.147 | -0.019 | 0.922 | -0.007 | 0.972 | -0.052 | 0.794 | 0.038 | 0.843 | 0.153 | 0.428 | -0.044 | 0.820 |
| C16:1n-7 | 0.133 | 0.516 | -0.060 | 0.771 | -0.069 | 0.738 | -0.172 | 0.381 | -0.020 | 0.920 | -0.230 | 0.238 | 0.012 | 0.950 | 0.076 | 0.697 | -0.057 | 0.770 |
| C18:0 | 0.052 | 0.803 | 0.045 | 0.826 | 0.279 | 0.168 | 0.216 | 0.269 | -0.063 | 0.749 | 0.153 | 0.436 | -0.100 | 0.605 | -0.060 | 0.758 | -0.156 | 0.418 |
| C18:1n-9 | 0.262 | 0.196 | 0.103 | 0.615 | 0.253 | 0.213 | -0.329 | 0.087 | -0.020 | 0.920 | **-0.398** | **0.036** | 0.097 | 0.616 | -0.037 | 0.849 | 0.053 | 0.786 |
| C18:2n-6 | -0.182 | 0.374 | 0.146 | 0.478 | 0.009 | 0.965 | 0.161 | 0.413 | 0.147 | 0.457 | 0.151 | 0.443 | -0.035 | 0.858 | -0.037 | 0.850 | -0.154 | 0.424 |
| C18:3n-3 | 0.015 | 0.944 | 0.131 | 0.525 | 0.052 | 0.801 | 0.100 | 0.614 | 0.181 | 0.357 | 0.066 | 0.738 | -0.284 | 0.135 | -0.169 | 0.381 | -0.117 | 0.545 |
| C20:4n-6 | 0.347 | 0.082 | 0.112 | 0.585 | **0.426** | **0.030** | -0.169 | 0.391 | -0.003 | 0.987 | -0.053 | 0.787 | 0.120 | 0.537 | 0.377 | 0.044 | 0.229 | 0.232 |
| C20:5n-3 | **-0.605** | **0.001** | -0.253 | 0.213 | **-0.466** | **0.017** | -0.259 | 0.183 | -0.121 | 0.538 | -0.207 | 0.290 | 0.046 | 0.814 | -0.112 | 0.563 | 0.018 | 0.928 |
| C22:6n-3 | 0.094 | 0.646 | -0.077 | 0.709 | 0.276 | 0.172 | -0.264 | 0.174 | -0.273 | 0.159 | -0.294 | 0.129 | -0.084 | 0.665 | -0.157 | 0.417 | -0.126 | 0.513 |
| *Saturated FA* | 0.268 | 0.186 | 0.167 | 0.414 | 0.341 | 0.088 | 0.095 | 0.630 | -0.070 | 0.725 | 0.028 | 0.888 | 0.001 | 0.997 | 0.125 | 0.519 | -0.084 | 0.666 |
| *Monounsaturated FA* | 0.125 | 0.541 | 0.006 | 0.975 | 0.055 | 0.789 | **-0.471** | **0.011** | 0.017 | 0.931 | **-0.553** | **0.002** | 0.107 | 0.579 | -0.121 | 0.533 | 0.077 | 0.691 |
| *n-3 Polyunsaturated FA* | -0.266 | 0.189 | -0.145 | 0.481 | -0.085 | 0.678 | -0.293 | 0.130 | -0.225 | 0.250 | -0.276 | 0.155 | -0.066 | 0.735 | -0.156 | 0.420 | -0.092 | 0.634 |
| *n-6 Polyunsaturated FA* | 0.053 | 0.797 | 0.167 | 0.415 | 0.256 | 0.207 | 0.115 | 0.559 | 0.175 | 0.374 | 0.163 | 0.407 | 0.057 | 0.770 | 0.232 | 0.226 | -0.055 | 0.777 |
| *n*-6 : *n*-3 *Polyunsaturated FA* | 0.181 | 0.375 | 0.150 | 0.463 | 0.092 | 0.653 | 0.316 | 0.101 | 0.319 | 0.098 | 0.337 | 0.079 | 0.095 | 0.623 | 0.292 | 0.124 | 0.123 | 0.524 |

NOTE: Significant correlations (*P* < 0.05) are presented in bold.

Sum of saturated fatty acids (Saturated FA) =Σ (C12:0, C13:0, C14:0, C15:0, C16:0, C17:0, C18:0, C20:0, C22:0, C24:0)
Sum of Monounsaturated fatty acids (Monounsaturated FA) = Σ (C14:1n-5, C16:1n-9, C16:1n-7, C18:1n-9, C18:1n-7, C20:1n-7, C20:1n-9, C22:1n-9, C24:1n-9)

Sum of n-3 Polyunsaturated fatty acids (n-3 Polyunsaturated FA) = Σ (C18:3n-3, C20:3n-3, C20:4n-3, C20:5n-3, C22:5n-3, C22:6n-3)

Sum of n-6 Polyunsaturated fatty acids (n-6 Polyunsaturated FA) = Σ (C18:2n-6, C18:3n-6, C20:2n-6, C20:3n-6, C20:4n-6, C22:4n-6, C22:5n-6)
