# Supplementary material for: The fatty acid composition in follicles is related to the developmental potential of oocytes up to the blastocyst stage: a single-centre cohort study
Source: Reprod Biol Endocrinol. 2022 Jul 25;20:107. doi: 10.1186/s12958-022-00974-7 (PMC9310456; doi:10.1186/s12958-022-00974-7)
Supplement: Supplementary file 2 — Additional file 2. Correlation analysis for proportions (% by weight) of specific FF FA and FA groups with embryo outcome among overweight/obese women (n = 54) in 3 age groups. [file 12958_2022_974_MOESM2_ESM.docx]

**Additional File 2. Correlation analysis for proportions (% by weight) of specific FF FA and FA groups with embryo outcome among overweight/obese**

**women (n = 54) in 3 age groups.**

| **Items** | **20-30 years** | | | | | | **31-34 years** | | | | | | **≥ 35 years** | | | | | |
| --- | --- | --- | --- | --- | --- | --- | --- | --- | --- | --- | --- | --- | --- | --- | --- | --- | --- | --- |
|  | **ED3-5/2PN** | | **EB5/2PN** | | **EUR** | | **ED3-5/2PN** | | **EB5/2PN** | | **EUR** | | **ED3-5/2PN** | | **EB5/2PN** | | **EUR** | |
| **Fatty acid (FA)** | r | *P*-value | r | *P*-value | r | *P*-value | r | *P*-value | r | *P*-value | r | *P*-value | r | *P*-value | r | *P*-value | r | *P*-value |
| C16:0 | -0.316 | 0.142 | -0.315 | 0.144 | -0.236 | 0.277 | -0.125 | 0.656 | 0.478 | 0.072 | -0.064 | 0.820 | 0.371 | 0.158 | 0.285 | 0.284 | 0.485 | 0.057 |
| C16:1n-7 | 0.157 | 0.474 | -0.026 | 0.907 | 0.182 | 0.406 | 0.151 | 0.590 | 0.370 | 0.174 | 0.164 | 0.560 | 0.247 | 0.356 | -0.157 | 0.562 | 0.287 | 0.281 |
| C18:0 | 0.167 | 0.447 | 0.090 | 0.685 | -0.150 | 0.493 | **-0.642** | **0.010** | -0.213 | 0.446 | **-0.630** | **0.012** | -0.165 | 0.541 | 0.125 | 0.644 | -0.420 | 0.106 |
| C18:1n-9 | 0.287 | 0.184 | 0.134 | 0.543 | 0.137 | 0.533 | -0.128 | 0.649 | 0.127 | 0.652 | -0.065 | 0.819 | **0.824** | **<0.001** | 0.281 | 0.293 | **0.770** | **<0.001** |
| C18:2n-6 | -0.074 | 0.736 | 0.079 | 0.719 | -0.154 | 0.484 | -0.132 | 0.638 | 0.085 | 0.762 | -0.227 | 0.417 | -0.177 | 0.512 | 0.254 | 0.342 | -0.311 | 0.240 |
| C18:3n-3 | 0.384 | 0.071 | 0.376 | 0.077 | 0.303 | 0.160 | 0.060 | 0.832 | 0.413 | 0.126 | 0.058 | 0.836 | 0.096 | 0.724 | -0.045 | 0.868 | 0.045 | 0.868 |
| C20:4n-6 | 0.085 | 0.700 | 0.039 | 0.858 | 0.150 | 0.494 | -0.320 | 0.245 | -0.132 | 0.640 | -0.203 | 0.468 | 0.195 | 0.468 | 0.036 | 0.895 | 0.147 | 0.587 |
| C20:5n-3 | -0.244 | 0.261 | -0.190 | 0.386 | -0.280 | 0.196 | -0.269 | 0.333 | -0.063 | 0.823 | -0.058 | 0.838 | -0.285 | 0.285 | -0.442 | 0.086 | -0.135 | 0.619 |
| C22:6n-3 | -0.305 | 0.156 | -0.282 | 0.193 | -0.244 | 0.261 | -0.215 | 0.442 | -0.111 | 0.693 | -0.023 | 0.936 | -0.336 | 0.203 | -0.234 | 0.384 | -0.233 | 0.385 |
| *Saturated FA* | -0.215 | 0.323 | -0.283 | 0.191 | -0.296 | 0.171 | -0.447 | 0.095 | 0.273 | 0.325 | -0.374 | 0.169 | 0.189 | 0.482 | 0.256 | 0.339 | 0.147 | 0.586 |
| *Monounsaturated FA* | 0.187 | 0.394 | 0.029 | 0.895 | 0.105 | 0.634 | 0.230 | 0.411 | 0.064 | 0.820 | 0.250 | 0.369 | **0.555** | **0.026** | -0.064 | 0.813 | **0.656** | **0.006** |
| *n-3 Polyunsaturated FA* | -0.227 | 0.297 | -0.213 | 0.329 | -0.229 | 0.294 | -0.223 | 0.424 | -0.040 | 0.887 | -0.027 | 0.923 | -0.312 | 0.239 | -0.356 | 0.176 | -0.190 | 0.481 |
| *n-6 Polyunsaturated FA* | -0.058 | 0.792 | 0.033 | 0.883 | -0.127 | 0.563 | -0.237 | 0.396 | 0.061 | 0.830 | -0.299 | 0.279 | -0.072 | 0.790 | 0.324 | 0.220 | -0.279 | 0.295 |
| *n*-6 : *n*-3 *Polyunsaturated FA* | 0.225 | 0.301 | 0.292 | 0.176 | 0.237 | 0.276 | 0.069 | 0.807 | 0.075 | 0.789 | -0.152 | 0.589 | 0.162 | 0.548 | 0.430 | 0.096 | -0.009 | 0.973 |

NOTE: Significant correlations (*P* < 0.05) are presented in bold.

Sum of saturated fatty acids (Saturated FA) =Σ (C12:0, C13:0, C14:0, C15:0, C16:0, C17:0, C18:0, C20:0, C22:0, C24:0)
Sum of Monounsaturated fatty acids (Monounsaturated FA) = Σ (C14:1n-5, C16:1n-9, C16:1n-7, C18:1n-9, C18:1n-7, C20:1n-7, C20:1n-9, C22:1n-9, C24:1n-9)

Sum of n-3 Polyunsaturated fatty acids (n-3 Polyunsaturated FA) = Σ (C18:3n-3, C20:3n-3, C20:4n-3, C20:5n-3, C22:5n-3, C22:6n-3)

Sum of n-6 Polyunsaturated fatty acids (n-6 Polyunsaturated FA) = Σ (C18:2n-6, C18:3n-6, C20:2n-6, C20:3n-6, C20:4n-6, C22:4n-6, C22:5n-6)
