# Supplementary material for: The fatty acid composition in follicles is related to the developmental potential of oocytes up to the blastocyst stage: a single-centre cohort study
Source: Reprod Biol Endocrinol. 2022 Jul 25;20:107. doi: 10.1186/s12958-022-00974-7 (PMC9310456; doi:10.1186/s12958-022-00974-7)
Supplement: Supplementary file 3 — Additional file 3: Supplementary 1. Comparison of the proportions of specific FF FA and FA groups in free fatty acids (FFA), phospholipids (PL), cholesterol esters (CHE) and triglycerides (TG)) of the least (1st quartile, Q1) and best (4th quartile, Q4) performing quartiles, based on the assisted reproductive technology outcome parameters related to embryos developing between days 3 and 5 relative to oocytes with two pronuclei (ED3-5/2PN), expanded blastocysts on day 5 relative to oocytes with two pronuclei (EB5/2PN) and embryo utilization rate (EUR). Means or Median of parameters which show significant differences (P < 0.05) or a trend (0.05 ≤ P < 0.10) are presented in bold. Supplementary Table 2. Concentration of TFA or FA families and relative proportions of individual FF FA of normal weight, overweight and obese women. Supplementary Table 3. Concentration of TFA or FA families and relative proportions of individual FF FA in three age groups. [file 12958_2022_974_MOESM3_ESM.docx]

**Supplementary 1. Comparison of the proportions of specific FF FA and FA groups in free fatty acids (FFA), phospholipids (PL), cholesterol esters (CHE) and triglycerides (TG)) of the least (1st quartile, Q1) and best (4th quartile, Q4) performing quartiles, based on the assisted reproductive technology outcome parameters related to embryos developing between days 3 and 5 relative to oocytes with two pronuclei (ED3-5/2PN), expanded blastocysts on day 5 relative to oocytes with two pronuclei (EB5/2PN) and embryo utilization rate (EUR). Means or Median of parameters which show significant differences (*P* < 0.05) or a trend (0.05** ≤ ***P* < 0.10) are presented in bold.**

| **Items** | **ED3-5/2PN** | | | | **EB5/2PN** | | | | **EUR** | | | |
| --- | --- | --- | --- | --- | --- | --- | --- | --- | --- | --- | --- | --- |
| FFA, % by weight | **Q1** | **Q4** | **Statistics^1^** | ***P*-value** | **Q1** | **Q4** | **Statistics^1^** | ***P*-value** | **Q1** | **Q4** | **Statistics^1^** | ***P*-value** |
| C16:0 | 25.8 | 25.6 | T | 0.547 | 25.6 | 25.6 | T | 0.947 | 25.5 | 25.4 | T | 0.874 |
| C16:1n-7 | 2.46 | 2.47 | T-log | 0.983 | 2.16 | 2.50 | MW | 0.122 | 2.22 | 2.39 | MW | 0.238 |
| C18:0 | 10.8 | 10.7 | MW | 0.970 | 11.1 | 11.2 | T-log | 0.847 | 10.8 | 10.6 | MW | 0.616 |
| C18:1n-9 | 26.5 | 27.5 | T | 0.185 | 27.1 | 27.8 | T | 0.312 | 26.9 | 28.0 | T | 0.141 |
| C18:2n-6 | 16.1 | 15.8 | T | 0.652 | 15.7 | 16.1 | MW | 0.962 | 16.2 | 16.1 | T | 0.803 |
| C18:3n-3 | 0.51 | 0.57 | MW | 0.229 | 0.50 | 0.57 | MW | 0.161 | 0.53 | 0.57 | MW | 0.475 |
| C20:4n-6 | 1.54 | 1.60 | T | 0.671 | **1.44** | **1.65** | T | **0.090** | **1.46** | **1.71** | T | **0.062** |
| C20:5n-3 | **0.21** | **0.15** | T-log | **0.017** | 0.16 | 0.17 | T-log | 0.984 | 0.18 | 0.16 | T-log | 0.501 |
| C22:6n-3 | **1.07** | **0.89** | T | **0.057** | 0.98 | 0.88 | T | 0.275 | 1.00 | 0.93 | T | 0.446 |
| *Saturated FA* | 41.5 | 40.7 | T | 0.299 | 40.8 | 40.7 | T | 0.847 | 41.1 | 40.0 | T | 0.188 |
| *Monounsaturated FA* | 33.3 | 34.1 | T | 0.321 | 33.5 | 34.4 | T | 0.307 | 33.5 | 34.6 | T | 0.225 |
| *n-3 Polyunsaturated FA* | 2.32 | 2.09 | T-log | 0.101 | 2.32 | 2.22 | T | 0.502 | 2.34 | 2.20 | T | 0.329 |
| *n-6 Polyunsaturated FA* | 19.7 | 19.5 | T | 0.784 | 19.4 | 20.1 | MW | 0.706 | 19.8 | 19.9 | T | 0.851 |
| *n*-6 : *n*-3 *Polyunsaturated FA* | 8.63 | 9.56 | T | 0.103 | 8.99 | 9.36 | T | 0.542 | 8.83 | 9.43 | T | 0.262 |
| PL, % by weight | **Q1** | **Q4** | **Statistics^1^** | ***P*-value** | **Q1** | **Q4** | **Statistics^1^** | ***P*-value** | **Q1** | **Q4** | **Statistics^1^** | ***P*-value** |
| C16:0 | 30.5 | 30.2 | T-log | 0.420 | 30.4 | 30.1 | T-log | 0.54 | 30.3 | 30.1 | T | 0.720 |
| C16:1n-7 | 0.34 | 0.37 | MW | 0.377 | 0.34 | 0.37 | MW | 0.361 | 0.32 | 0.37 | MW | 0.440 |
| C18:0 | 14.0 | 13.9 | T | 0.859 | 14.1 | 13.8 | T | 0.160 | 14.1 | 13.9 | T | 0.440 |
| C18:1n-9 | 9.54 | 9.62 | T | 0.782 | 9.63 | 9.50 | MW | 0.172 | 9.59 | 9.26 | T | 0.211 |
| C18:2n-6 | 17.4 | 17.7 | T-log | 0.609 | 17.5 | 18.1 | T | 0.315 | 17.9 | 17.6 | T | 0.623 |
| C18:3n-3 | 0.18 | 0.20 | T-log | 0.245 | **0.18** | **0.21** | T-log | **0.084** | 0.18 | 0.20 | T-log | 0.365 |
| C20:4n-6 | 9.72 | 10.4 | T | 0.164 | 9.92 | 10.7 | T | 0.108 | **9.82** | **11.1** | T | **0.007** |
| C20:5n-3 | **0.85** | **0.63** | T-log | **0.025** | **0.77** | **0.59** | T-log | **0.021** | **0.77** | **0.63** | T-log | **0.079** |
| C22:6n-3 | **4.12** | **3.59** | T | **0.058** | **3.99** | **3.62** | T | **0.089** | 3.99 | 3.81 | T | 0.458 |
| *Saturated FA* | 46.6 | 45.9 | T | 0.166 | **46.6** | **45.7** | T | **0.058** | 46.4 | 45.8 | T | 0.227 |
| *Monounsaturated FA* | 12.2 | 12.3 | T | 0.983 | 12.4 | 12.2 | T | 0.693 | 12.3 | 11.8 | T | 0.156 |
| *n-3 Polyunsaturated FA* | **6.37** | **5.50** | T | **0.043** | **6.06** | **5.47** | T | **0.070** | 6.08 | 5.71 | T | 0.283 |
| *n-6 Polyunsaturated FA* | **31.6** | **33.0** | T | **0.012** | **32.0** | **33.4** | T | **0.017** | **32.1** | **33.4** | T | **0.025** |
| *n*-6 : *n*-3 *Polyunsaturated FA* | **5.17** | **6.22** | T-log | **0.010** | **5.42** | **6.23** | T-log | **0.014** | 5.44 | 5.99 | T-log | 0.110 |
| CHE, % by weight | **Q1** | **Q4** | **Statistics^1^** | ***P*-value** | **Q1** | **Q4** | **Statistics^1^** | ***P*-value** | **Q1** | **Q4** | **Statistics^1^** | ***P*-value** |
| C16:0 | 12.0 | 12.0 | T | 0.951 | 11.8 | 11.8 | T-log | 0.984 | 11.8 | 12.0 | T | 0.385 |
| C16:1n-7 | 1.82 | 1.89 | T-log | 0.705 | 1.86 | 1.78 | T-log | 0.67 | 1.89 | 1.87 | T-log | 0.900 |
| C18:0 | 0.90 | 0.86 | T | 0.215 | 0.86 | 0.84 | T-log | 0.496 | 0.86 | 0.86 | T-log | 0.987 |
| C18:1n-9 | 20.5 | 20.6 | T | 0.939 | 20.6 | 20.4 | T | 0.766 | 20.2 | 20.1 | T-log | 0.902 |
| C18:2n-6 | 48.0 | 48.3 | T | 0.802 | 47.8 | 48.6 | T | 0.440 | 48.1 | 47.9 | T | 0.827 |
| C18:3n-3 | 0.43 | 0.46 | T | 0.434 | 0.45 | 0.45 | T | 0.912 | 0.44 | 0.46 | T | 0.472 |
| C20:4n-6 | 7.11 | 7.65 | T | 0.222 | 7.37 | 7.83 | T | 0.243 | **7.02** | **8.16** | T-log | **0.004** |
| C20:5n-3 | 0.60 | 0.58 | MW | 0.233 | **0.68** | **0.58** | MW | **0.071** | 0.60 | 0.62 | MW | 0.521 |
| C22:6n-3 | 0.55 | 0.50 | T | 0.266 | 0.52 | 0.51 | T | 0.702 | 0.51 | 0.53 | T | 0.593 |
| *Saturated FA* | 14.4 | 14.3 | T | 0.770 | 14.3 | 14.1 | T | 0.481 | 14.1 | 14.2 | MW | 0.521 |
| *Monounsaturated FA* | 24.5 | 24.0 | MW | 0.634 | 24.6 | 24.2 | T | 0.589 | 23.8 | 23.6 | MW | 0.608 |
| *n-3 Polyunsaturated FA* | **2.05** | **1.71** | **MW** | **0.073** | 1.90 | 1.73 | MW | 0.175 | 1.96 | 1.77 | MW | 0.238 |
| *n-6 Polyunsaturated FA* | 56.6 | 57.6 | MW | 0.316 | 56.4 | 57.7 | T | 0.171 | 56.5 | 57.5 | T | 0.329 |
| *n*-6 : *n*-3 *Polyunsaturated FA* | **27.8** | **33.8** | MW | **0.049** | 29.9 | 32.3 | MW | 0.125 | 30.4 | 32.7 | MW | 0.204 |
| TG, % by weight | **Q1** | **Q4** | **Statistics^1^** | ***P*-value** | **Q1** | **Q4** | **Statistics^1^** | ***P*-value** | **Q1** | **Q4** | **Statistics^1^** | ***P*-value** |
| C16:0 | **22.1** | **23.6** | T | **0.018** | 22.7 | 23.1 | T | 0.608 | **22.0** | **23.6** | **T** | **0.023** |
| C16:1n-7 | 2.37 | 2.76 | MW | 0.202 | 2.32 | 2.62 | MW | 0.180 | 2.30 | 2.43 | MW | 0.198 |
| C18:0 | 3.18 | 3.15 | MW | 0.854 | 3.16 | 3.18 | MW | 0.899 | 3.18 | 3.24 | MW | 0.624 |
| C18:1n-9 | 38.5 | 39.3 | MW | 0.883 | 38.6 | 39.3 | MW | 0.556 | 38.9 | 38.9 | MW | 0.797 |
| C18:2n-6 | 15.4 | 14.9 | T-log | 0.502 | 14.9 | 14.9 | T-log | 0.991 | 15.6 | 15.8 | MW | 0.835 |
| C18:3n-3 | 0.89 | 0.87 | T-log | 0.821 | 0.80 | 0.87 | MW | 0.204 | 0.92 | 0.94 | T-log | 0.779 |
| C20:4n-6 | 1.78 | 1.65 | T | 0.438 | 1.68 | 1.54 | T-log | 0.330 | 1.67 | 1.65 | T-log | 0.887 |
| C20:5n-3 | **0.22** | **0.13** | T-log | **0.009** | **0.21** | **0.14** | T-log | **0.011** | **0.22** | **0.14** | T-log | **0.016** |
| C22:6n-3 | **0.41** | **0.30** | T-log | **0.029** | **0.44** | **0.33** | T | **0.007** | **0.43** | **0.36** | T | **0.094** |
| *Saturated FA* | **28.6** | **30.9** | MW | **0.030** | 29.9 | 30.0 | MW | 0.611 | 28.6 | 30.1 | MW | 0.111 |
| *Monounsaturated FA* | 45.9 | 46.2 | MW | 0.980 | 44.9 | 45.8 | T | 0.219 | 45.4 | 45.7 | MW | 0.893 |
| *n-3 Polyunsaturated FA* | **2.03** | **1.66** | T-log | **0.026** | 1.94 | 1.77 | T-log | 0.311 | 2.03 | 1.83 | T-log | 0.210 |
| *n-6 Polyunsaturated FA* | 18.7 | 17.9 | T-log | 0.324 | 18.2 | 18.0 | T-log | 0.773 | 19.3 | 19.1 | MW | 0.425 |
| *n*-6 : *n*-3 *Polyunsaturated FA* | **9.21** | **10.8** | **T-log** | **0.048** | 9.40 | 10.2 | T-log | 0.296 | 9.81 | 9.49 | MW | 0.854 |

NOTE: Mean, in case of normal data distribution or Median, in case of non-normal data distribution, Parameters which show significant differences (*P* < 0.05) or a trend (0.05 ≤ *P* < 0.10) are presented in bold.

**^1^** Statistics: MW: Mann-Whitney U tests; T: independent T-test; T-log: a T-test has been performed when log-transformed (T-log) data were normally distributed. For log-transformed data (T-log), log-back transformations were carried out to calculate the geometric mean.

Sum of saturated fatty acids (Saturated FA) =Σ (C12:0, C13:0, C14:0, C15:0, C16:0, C17:0, C18:0, C20:0, C22:0, C24:0)
Sum of Monounsaturated fatty acids (Monounsaturated FA) = Σ (C14:1n-5, C16:1n-9, C16:1n-7, C18:1n-9, C18:1n-7, C20:1n-7, C20:1n-9, C22:1n-9, C24:1n-9)

Sum of n-3 Polyunsaturated fatty acids (n-3 Polyunsaturated FA) = Σ (C18:3n-3, C20:3n-3, C20:4n-3, C20:5n-3, C22:5n-3, C22:6n-3)

Sum of n-6 Polyunsaturated fatty acids (n-6 Polyunsaturated FA) = Σ (C18:2n-6, C18:3n-6, C20:2n-6, C20:3n-6, C20:4n-6, C22:4n-6, C22:5n-6)

**Supplementary Table 2. Concentration of TFA or FA families and relative proportions of individual FF FA of normal weight, overweight and obese women.**

| **Fatty acid (FA)** | **Statistics^1^** | **Mean or Median** | | | **95% CI or IQR** | | |
| --- | --- | --- | --- | --- | --- | --- | --- |
|  |  | **Normal weight**  **(n=83)** | **Overweight**  **(n=37)** | **Obese**  **(n=17)** | **Normal weight** | **Overweight** | **Obese** |
| **Concentration, µM** |  |  |  |  |  |  |  |
| *Total FA* | A | **2205^a^** | **2506^b^** | **2587^b^** | 2046-2363 | 2307-2705 | 2199-2976 |
| *Saturated FA* | A | **733^a^** | **842^b^** | **880^b^** | 679-788 | 773-910 | 741-1020 |
| *Monounsaturated FA* | A | **469^a^** | **538^b^** | **563^b^** | 433-504 | 489-588 | 475-651 |
| *n-3 Polyunsaturated FA* | A-log | 79.0 | 89.2 | 82.8 | 72.5-86.1 | 80.8-98.4 | 72.6-94.4 |
| *n-6 Polyunsaturated FA* | A | **755^a^** | **866^b^** | **901^b^** | 699-812 | 786-945 | 743-1058 |
| **Proportions, % by weight** |  |  |  |  |  |  |  |
| *Saturated FA* | A | 31.3 | 31.6 | 31.9 | 30.9-31.7 | 31.0-32.1 | 31.1-32.6 |
| C12:0 | A-log | 0.10 | 0.12 | 0.12 | 0.09-0.12 | 0.10-0.15 | 0.09-0.15 |
| C13:0 | A-log | 0.50 | 0.46 | 0.44 | 0.47-0.54 | 0.43-0.50 | 0.39-0.49 |
| C14:0 | A-log | 0.58 | 0.55 | 0.62 | 0.55-0.61 | 0.50-0.61 | 0.56-0.68 |
| C15:0 | K | 0.53 | 0.49 | 0.48 | 0.41-0.58 | 0.40-0.62 | 0.36-0.52 |
| C16:0 | A | **20.6^a^** | **21.1^ab^** | **21.6^b^** | 20.3-20.9 | 20.6-21.6 | 20.8-22.3 |
| C17:0 | A | **0.26^a^** | **0.24^b^** | **0.23^b^** | 0.25-0.27 | 0.22-0.25 | 0.21-0.25 |
| C18:0 | K | 8.18 | 8.30 | 8.09 | 7.79-8.70 | 7.79-8.82 | 7.66-8.57 |
| C20:0 | A-log | 0.10 | 0.09 | 0.08 | 0.09-0.11 | 0.08-0.10 | 0.06-0.10 |
| C22:0 | A | **0.09^a^** | **0.08^ab^** | **0.07^b^** | 0.08-0.10 | 0.06-0.09 | 0.05-0.09 |
| C24:0 | K | 0.04 | 0.03 | 0.04 | 0.02-0.06 | 0.03-0.04 | 0.03-0.04 |
| *Monounsaturated FA* | K | 21.0 | 21.3 | 22.8 | 20.0-22.5 | 20.3-22.9 | 20.7-23.1 |
| C14:1n-5 | A | 0.15 | 0.14 | 0.13 | 0.14-0.17 | 0.11-0.17 | 0.09-0.18 |
| C 16:1n-7 | K | **0.99^a^** | **1.14^ab^** | **1.41^b^** | 0.83-1.25 | 1.00-1.33 | 1.23-1.52 |
| C16:1n-9 | K | 1.78 | 1.55 | 1.74 | 1.02-2.62 | 0.90-2.29 | 0.95-2.26 |
| C18:1n-7 | A | 1.44 | 1.47 | 1.40 | 1.40-1.48 | 1.40-1.54 | 1.30-1.51 |
| C18:1n-9 | A | 16.2 | 16.6 | 16.7 | 15.8-16.6 | 16.0-17.2 | 15.8-17.7 |
| C20:1n-7 + C20:1n-9 | K | 0.33 | 0.32 | 0.31 | 0.07-0.40 | 0.29-0.36 | 0.09-0.34 |
| C22:1n-9 | K | 0.04 | 0.04 | 0.04 | 0.03-0.07 | 0.04-0.06 | 0.03-0.08 |
| C24:1n-9 | K | 0.03 | 0.00 | 0.00 | 0.00-0.06 | 0.00-0.04 | 0.00-0.05 |
| *n-3 Polyunsaturated FA* | A-log | **4.35^a^** | **4.18^ab^** | **3.81^b^** | 4.12-4.58 | 3.86-4.53 | 3.53-4.10 |
| C18:3n-3 | A-log | 0.37 | 0.40 | 0.39 | 0.35-0.40 | 0.36-0.44 | 0.33-0.46 |
| C20:3n-3 | A-log | 0.02 | 0.02 | 0.02 | 0.02-0.02 | 0.02-0.02 | 0.01-0.02 |
| C20:4n-3 | K | **0.26^a^** | **0.21^ab^** | **0.17^b^** | 0.19-0.35 | 0.18-0.27 | 0.16-0.24 |
| C20:5n-3 | K | 0.60 | 0.54 | 0.51 | 0.45-0.85 | 0.43-0.84 | 0.32-0.64 |
| C22:5n-3 | A-log | **0.51^a^** | **0.49^ab^** | **0.45^b^** | 0.49-0.54 | 0.45-0.53 | 0.42-0.49 |
| C22:6n-3 | A-log | 2.43 | 2.39 | 2.22 | 2.30-2.57 | 2.18-2.62 | 2.06-2.38 |
| *n-6 Polyunsaturated FA* | K | 35.6 | 36.2 | 36.2 | 33.3-36.8 | 33.1-37.6 | 33.1-37.1 |
| C18:2n-6 | A | 24.5 | 23.9 | 24.0 | 23.9-25.1 | 22.9-25.0 | 22.0-26.0 |
| C18:3n-6 | K | 0.12 | 0.15 | 0.15 | 0.10-0.16 | 0.12-0.19 | 0.12-0.23 |
| C20:2n-6 | K | 0.31 | 0.31 | 0.30 | 0.28-0.36 | 0.26-0.35 | 0.28-0.36 |
| C20:3n-6 | A | **1.91^a^** | **2.20^b^** | **2.38^b^** | 1.81-2.01 | 2.08-2.33 | 2.17-2.58 |
| C20:4n-6 | A | 7.83 | 8.35 | 8.32 | 7.53-8.14 | 7.89-8.82 | 7.77-8.88 |
| C22:4n-6 | A-log | 0.34 | 0.34 | 0.37 | 0.33-0.36 | 0.32-0.36 | 0.33-0.41 |
| C22:5n-6 | A-log | **0.16^a^** | **0.17^ab^** | **0.20^b^** | 0.14-0.17 | 0.16-0.19 | 0.17-0.23 |
| *n*-6 : *n*-3 *Polyunsaturated FA* | A-log | **8.09^a^** | **8.45^ab^** | **9.34^b^** | 7.65-8.56 | 7.79-9.17 | 8.32-10.5 |
| Unknown FA | A-log | 7.18 | 6.42 | 6.27 | 6.54-7.88 | 5.46-7.54 | 5.07-7.75 |

NOTE: Means or Median of parameters which show significant differences (*P* < 0.05) are presented in bold.

^1^ Statistics: A: ANOVA; A-log: ANOVA analysis has been performed when log-transformed data were normally distributed. For log transformed parameters (A-log), log-back transformations were carried out to calculate the geometric mean and 95% confidence interval (CI). ANOVA followed by Least Significant Difference test to determine significance between BMI groups at *P* < 0.05, which are indicated by superscripted letters (a, b). For non-normality distributed parameters (K), median and interquartile range (IQR) are presented. Kruskal Wallis test followed by a post-hoc Mann-Whitney-Wilcoxon test to determine significance between BMI groups at *P* < 0.05, which are indicated by superscripted letters (a, b).

Sum of saturated fatty acids (Saturated FA) =Σ (C12:0, C13:0, C14:0, C15:0, C16:0, C17:0, C18:0, C20:0, C22:0, C24:0).
Sum of Monounsaturated fatty acids (Monounsaturated FA) = Σ (C14:1n-5, C16:1n-9, C 16:1n-7, C18:1n-9, C18:1n-7, C20:1n-7, C20:1n-9, C22:1n-9, C24:1n-9).

Sum of n-3 Polyunsaturated fatty acids (n-3 Polyunsaturated FA) = Σ (C18:3n-3, C20:3n-3, C20:4n-3, C20:5n-3, C22:5n-3, C22:6n-3).

Sum of n-6 Polyunsaturated fatty acids (n-6 Polyunsaturated FA) = Σ (C18:2n-6, C18:3n-6, C20:2n-6, C20:3n-6, C20:4n-6, C22:4n-6, C22:5n-6).

**Supplementary Table 3. Concentration of TFA or FA families and relative proportions of individual FF FA in three age groups.**

| **Fatty acid (FA)** | **Statistics^1^** | **Mean or Median** | | | **95% CI or IQR** | | |
| --- | --- | --- | --- | --- | --- | --- | --- |
|  |  | **20-30 years**  **(n = 49)** | **31-34 years**  **(n = 43)** | **35-42 years**  **(n = 45)** | **20-30 years** | **31-34 years** | **35-42 years** |
| **Concentrations, µM** |  |  |  |  |  |  |  |
| *Total FA* | K | 2519 | 2197 | 2240 | 2057-2781 | 1610-2875 | 1528-3076 |
| *Saturated FA* | K | 844 | 735 | 736 | 660-939 | 515-960 | 546-1061 |
| *Monounsaturated FA* | K | 542 | 493 | 475 | 444-604 | 356-642 | 330-646 |
| *n-3 Polyunsaturated FA* | K | 80.6 | 85.8 | 79.1 | 73.9-96.5 | 62.8-106 | 63.8-108 |
| *n-6 Polyunsaturated FA* | K | 864 | 785 | 769 | 680-1008 | 519-1040 | 505-1009 |
| **Proportion, % by weight** |  |  |  |  |  |  |  |
| *Saturated FA* | A | 31.2 | 31.3 | 31.7 | 30.7-31.7 | 30.8-31.8 | 31.2-32.3 |
| C12:0 | K | 0.12 | 0.12 | 0.11 | 0.08-0.19 | 0.06-0.19 | 0.08-0.18 |
| C13:0 | K | 0.45 | 0.46 | 0.50 | 0.37-0.51 | 0.39-0.57 | 0.41-0.65 |
| C14:0 | A-log | **0.54^a^** | **0.55^a^** | **0.64^b^** | 0.51-0.58 | 0.51-0.60 | 0.60-0.69 |
| C15:0 | K | **0.52^ab^** | **0.54^a^** | **0.46^b^** | 0.38-0.58 | 0.48-0.59 | 0.40-0.54 |
| C16:0 | A | 20.8 | 20.8 | 21.1 | 20.4-21.2 | 20.4-21.2 | 20.6-21.6 |
| C17:0 | A-log | **0.23^a^** | **0.24^ab^** | **0.26^b^** | 0.22-0.24 | 0.23-0.26 | 0.25-0.27 |
| C18:0 | K | 8.19 | 8.16 | 8.15 | 7.88-8.70 | 7.68-8.64 | 7.71-8.81 |
| C20:0 | A-log | 0.09 | 0.10 | 0.09 | 0.08-0.10 | 0.09-0.12 | 0.08-0.11 |
| C22:0 | K | 0.08 | 0.09 | 0.08 | 0.07-0.10 | 0.05-0.11 | 0.05-0.12 |
| C24:0 | K | 0.04 | 0.04 | 0.04 | 0.03-0.05 | 0.02-0.05 | 0.03-0.06 |
| *Monounsaturated FA* | K | 20.6 | 21.5 | 21.3 | 19.7-22.9 | 20.6-22.6 | 20.0-22.9 |
| C14:1n-5 | K | 0.14 | 0.18 | 0.15 | 0.05-0.20 | 0.10-0.22 | 0.06-0.23 |
| C16:1n-7 | A-log | 1.06 | 1.08 | 1.20 | 0.97-1.15 | 0.98-1.18 | 1.08-1.34 |
| C16:1n-9 | K | 1.81 | 1.29 | 1.77 | 0.97-2.44 | 0.78-2.44 | 1.15-2.54 |
| C18:1n-7 | A-log | 1.43 | 1.47 | 1.39 | 1.38-1.49 | 1.41-1.52 | 1.33-1.46 |
| C18:1n-9 | A | 16.2 | 16.7 | 16.2 | 15.7-16.8 | 16.2-17.3 | 15.7-16.7 |
| C20:1n-7 + C20:1n-9 | K | 0.32 | 0.33 | 0.32 | 0.27-0.37 | 0.10-0.43 | 0.17-0.39 |
| C22:1n-9 | K | **0.04^a^** | **0.04^b^** | **0.04^ab^** | 0.04-0.08 | 0.03-0.05 | 0.03-0.08 |
| C24:1n-9 | K | 0.00 | 0.04 | 0.00 | 0.00-0.06 | 0.00-0.06 | 0.00-0.05 |
| *n-3 Polyunsaturated FA* | A-log | **3.99^a^** | **4.36^ab^** | **4.38^b^** | 3.77-4.22 | 4.05-4.69 | 4.05-4.74 |
| C18:3n-3 | A-log | 0.39 | 0.38 | 0.38 | 0.37-0.42 | 0.34-0.41 | 0.35-0.41 |
| C20:3n-3 | A-log | 0.02 | 0.02 | 0.02 | 0.02-0.02 | 0.02-0.02 | 0.02-0.02 |
| C20:4n-3 | K | 0.20 | 0.25 | 0.23 | 0.17-0.28 | 0.19-0.36 | 0.17-0.32 |
| C20:5n-3 | K | **0.48^a^** | **0.61^ab^** | **0.64^b^** | 0.40-0.63 | 0.45-0.79 | 0.48-1.00 |
| C22:5n-3 | A-log | 0.49 | 0.51 | 0.49 | 0.46-0.52 | 0.48-0.55 | 0.46-0.53 |
| C22:6n-3 | A-log | 2.27 | 2.49 | 2.45 | 2.13-2.42 | 2.30-2.69 | 2.25-2.66 |
| *n-6 Polyunsaturated FA* | A^2^ | **36.0^a^** | **35.5^ab^** | **34.5^b^** | 35.2-36.8 | 34.6-36.5 | 33.7-35.3 |
| C18:2n-6 | A | 24.7 | 24.5 | 23.6 | 23.9-25.4 | 23.5-25.5 | 22.7-24.6 |
| C18:3n-6 | K | 0.12 | 0.13 | 0.15 | 0.10-0.16 | 0.10-0.17 | 0.11-0.20 |
| C20:2n-6 | A-log | 0.31 | 0.33 | 0.32 | 0.29-0.33 | 0.31-0.35 | 0.30-0.34 |
| C20:3n-6 | A | 2.08 | 2.00 | 2.05 | 1.95-2.22 | 1.86-2.14 | 1.91-2.19 |
| C20:4n-6 | A | 8.27 | 7.98 | 7.84 | 7.91-8.63 | 7.57-8.40 | 7.38-8.29 |
| C22:4n-6 | A-log | 0.35 | 0.34 | 0.35 | 0.33-0.37 | 0.32-0.36 | 0.32-0.37 |
| C22:5n-6 | K | 0.19 | 0.16 | 0.15 | 0.14-0.23 | 0.13-0.18 | 0.13-0.21 |
| *n*-6 : *n*-3 *Polyunsaturated FA* | A-log | **9.01^a^** | **8.12^b^** | **7.85^b^** | 8.52-9.53 | 7.48-8.81 | 7.22-8.54 |
| Unknown FA | A-log | 6.72 | 6.55 | 7.29 | 5.80-7.80 | 5.75-7.45 | 6.51-8.17 |

NOTE: Means or Median of parameters which show significant differences (*P* < 0.05) are presented in bold.

^1^ Statistics: A: ANOVA; A-log: ANOVA analysis has been performed when log-transformed data were normally distributed. For log transformed parameters (A-log), log-back transformations were carried out to calculate the geometric mean and 95% confidence interval (CI). ANOVA followed by Least Significant Difference test to determine significance between BMI groups at *P* < 0.05, which are indicated by superscripted letters (a, b). For non-normality distributed parameters (K), median and interquartile range (IQR) are presented. Kruskal Wallis test followed by a post-hoc Mann-Whitney-Wilcoxon test to determine significance between age groups at *P* < 0.05, which are indicated by superscripted letters (a, b).

Sum of saturated fatty acids (Saturated FA) =Σ (C12:0, C13:0, C14:0, C15:0, C16:0, C17:0, C18:0, C20:0, C22:0, C24:0)
Sum of Monounsaturated fatty acids (Monounsaturated FA) = Σ (C14:1n-5, C16:1n-9, C 16:1n-7, C18:1n-9, C18:1n-7, C20:1n-7, C20:1n-9, C22:1n-9, C24:1n-9)

Sum of n-3 Polyunsaturated fatty acids (n-3 Polyunsaturated FA) = Σ (C18:3n-3, C20:3n-3, C20:4n-3, C20:5n-3, C22:5n-3, C22:6n-3)

Sum of n-6 Polyunsaturated fatty acids (n-6 Polyunsaturated FA) = Σ (C18:2n-6, C18:3n-6, C20:2n-6, C20:3n-6, C20:4n-6, C22:4n-6, C22:5n-6)
